# Pancreas as an Occult Source of Recurrent *Salmonella enteritidis* Bacteremia in an Immunocompromised Patient

**DOI:** 10.1155/2018/7296295

**Published:** 2018-06-11

**Authors:** Don Bambino Geno Tai, Laxmi Upadhyay, Ruchika Jain, Robert Goldstein

**Affiliations:** ^1^Montefiore New Rochelle Hospital, 16 Guion Place, New Rochelle, NY 10801, USA; ^2^Montefiore Medical Center, 1825 Eastchester Road, Bronx, NY 10461, USA

## Abstract

*Salmonella* infection usually presents as gastroenteritis and enteric fever. Some cases of bacteremia can lead to invasion of different organ systems and become occult sources for recurrence. Almost all organs of the body can be affected and have been reported in the literature. We report a rare case of repeated *Salmonella enteritidis* infection in a patient with systemic lupus erythematosus. She was treated with intravenous and oral antibiotics but still had recurrence of fevers and bacteremia. After a thorough workup, pancreatic abscesses were identified and drained with abscess culture revealing *Salmonella enteritidis*. She was then treated with a prolonged course of antibiotics and finally cleared the infection. This case demonstrates that nontyphoid *Salmonella* can be invasive and cause persistent infection. This is particularly pertinent in immunocompromised patients who are at an increased risk of infection. An aggressive workup and prolonged antibiotic course might be beneficial for this subset of patients.

## 1. Introduction

*Salmonella* species are Gram-negative, facultative anaerobic bacteria. *Salmonella* infection can present as gastroenteritis, enteric fever, bacteremia, a chronic carrier state, or localize to one or more sites [1]. *Salmonella* infection is mainly divided into typhoidal salmonellosis causing typhoid and paratyphoid fever, and nontyphoidal *Salmonella* (NTS) infection.

NTS infection usually presents as gastroenteritis and enteric fever. Occasionally, these infections lead to invasive disease. The majority of cases are caused by *Salmonella typhimurium* or *Salmonella enteritidis* [2]. Abscess formation or localization may happen after NTS bacteremia, frequently to the urinary system. Cardiothoracic, musculoskeletal, and central nervous system infections have also been reported [3]. Pancreatic abscess is rare.

## 2. Case Presentation

A 56-year-old female presented with repeated fevers in a span of four months. Her medical history was significant for systemic lupus erythematosus (SLE) with nephritis and hypothyroidism. Her medications were methylprednisolone and levothyroxine. She was allergic to cephalexin, penicillin, and levofloxacin. She was a nonsmoker, denied alcohol, and illicit drug use.

In her first hospital admission, she presented with fever and chills after traveling to Jamaica. She had no other symptoms. Her physical examination was only remarkable for fever of 38°C. Diagnostics revealed blood cultures positive for *Salmonella enteritidis*. Urine culture was negative. Due to her complicated history of antibiotic allergies, she was treated with aztreonam and discharged on trimethoprim/sulfamethoxazole (TMP/SMX) for two weeks.

She was well in the interim until one month later when she returned with fever (39°C), nausea, and dysuria. Physical examination identified costovertebral angle tenderness. Blood culture again grew the same organism, and urine culture was also positive. She was treated with aztreonam and discharged home with TMP/SMX for two weeks. Repeat urine culture as outpatient was negative for *Salmonella*.

Within the next month, she was readmitted with generalized body and joint pains. She was then treated with steroids for a lupus flare. During this admission, there were incidental findings of elevated lipase 668 U/L (reference range 4–66), and CT scan showed pancreatitis. She did not have any abdominal pain or symptoms to suggest acute pancreatitis. Her blood and urine cultures were negative. Gastroenterology service recommended that she be managed supportively. After clinical improvement, she was discharged to a rehabilitation facility.

In a week, she again developed fever accompanied by chills, nausea, and vomiting. There were no other symptoms. Physical examination was only remarkable for temperature of 38.5°C and blood pressure of 144/63 mm Hg. The examination was otherwise normal.

The serum white blood cell count was 13.1 × 10^3^ *µ*L (ref 4.8–10.8), and hemoglobin was of 7.9 (ref 12–16). Lipase was 22 U/L (ref 4–66). Bilirubins, transaminases, and the remainder of the metabolic profile were unremarkable. Blood and stool culture grew *Salmonella enteritidis* sensitive to ampicillin, aztreonam, ceftriaxone, ciprofloxacin, piperacillin/tazobactam, and trimethoprim/sulfamethoxazole.

**Figure 1:**
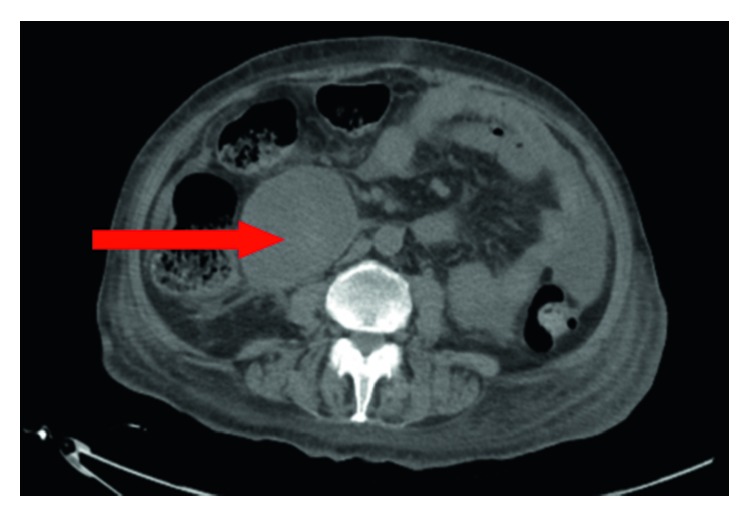
CT scan of the abdomen showing the mass near the pancreatic head.

Computed tomography (CT) scan of the abdomen showed enlargement of the pancreatic head with adjacent stranding consistent with pancreatitis along with adjacent low-density regions anteriorly and inferiorly consistent with inflammatory liquefaction (Figure 1). Other abdominal structures were normal. Magnetic resonance imaging (MRI) showed an 8 cm mass below the level of the pancreas.

She was initially given aztreonam for two weeks pending sampling of the pancreatic mass. The patient then changed her mind and refused the procedure. Since she was clinically doing well and repeat cultures were negative, she was monitored off antibiotics with a plan to do the procedure if she worsened.

While awaiting discharge, she had a fever of 38°C. Blood and stool cultures were again positive. This time, she was given intravenous ceftriaxone. Repeat CT and MRI demonstrated multiple complex fluid collections around the pancreas: a 9 × 6 cm mass inferior to the pancreas, a 7 × 4 cm mass superiorly, and two smaller masses abutting the head and body.

**Figure 2:**
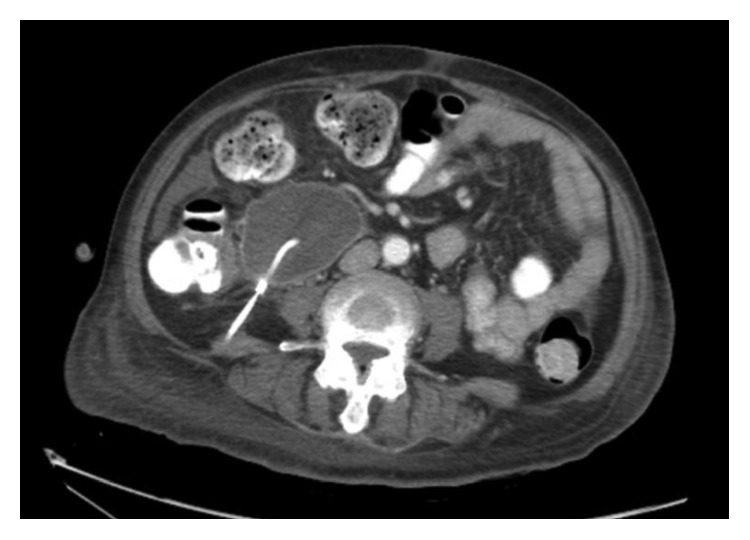
CT scan of the abdomen showing one of the pancreatic abscesses with drain placed.

CT-guided drainage was performed with purulent fluid identified. A Jackson-Pratt drain was then placed (Figure 2). Fluid culture grew *Salmonella enteritidis*. The patient was transitioned to 4 weeks of oral ciprofloxacin, which the patient tolerated despite the reported levofloxacin allergy. She was followed up as outpatient and was doing well.

## 3. Discussion

Recurrent *Salmonella* infection is a debilitating condition among patients with SLE. SLE in conjunction with immunosuppressant medications is a predisposing factor for *Salmonella* infection [4]. Recurrent bacteremia in lupus patients is partly due to defective immune function and immunodeficiency with steroid use [5].

NTS infection among SLE patients usually presents as gastroenteritis and enteric fever. It is uncommon for focal suppurations to occur, but there are reported cases involving joints/bones, lungs, urinary tract, and vascular system [6]. Several studies have reported pancreatic abscess from *Salmonella typhi* after episodes of pancreatitis [7]. Pancreatic abscess from nontyphoid *Salmonella* is more unusual. Five reports of pancreatic abscess were associated with *Salmonella typhimurium*, *Salmonella dublin*, and *Salmonella bovismorbificans* [8].

The frequency of clinical pancreatitis ranges from 28 to 62% in patients with *Salmonella* infection, while a prospective study by Pezzilli et al. suggests elevations in lipase are common in *Salmonella* infections but clinically insignificant [9, 10]. We identified only one other report of *Salmonella enteritidis* pancreatic abscess [11].

The proposed mechanisms of abscesses were hematogenous spread or direct invasion from the gastrointestinal tract. *Salmonella* can translocate from the gastrointestinal tract to damaged pancreatic tissues and was more frequent with *Salmonella typhi*. Bile invasion was also described but only during cases of obstruction with stones. An immunological mechanism was also proposed when lipopolysaccharide injection of *Salmonella typhi* resulted to pancreatic edema in animals. However, this mechanism was not fully understood [1]. These mechanisms were attributed to *Salmonella typhi* as it was more common in causing pancreatitis and abscess. However, it is unclear how similar the mechanisms are with nontyphoid *Salmonella* since it is rarer.

Our patient did not have infection during the hospitalization when she was incidentally found to have pancreatitis which was attributed to steroid use. One might argue that it was clinically insignificant and irrelevant to her course of recurrent bacteremia. It is possible that the bacteria seeded the pancreas creating a hidden nidus for repeated infection. Repeat cultures after antibiotics showed clearance after each episode. In the end, though, bacteremia recurrence resolved after abscess drainage and a prolonged course of antibiotics. Additionally, there was a change of antibiotic class which could have also contributed to final resolution.

A suppurative focus can be an occult source of recurrent infection [12]. A long duration of antibiotics and thorough search for a nidus are suggested in immunosuppressed patients with recurrent *Salmonella* bacteremia.
